# Gum arabic and red propolis protecteting colorectal preneoplastic lesions in a rat model of azoxymethane^1^

**DOI:** 10.1590/s0102-8650201900207

**Published:** 2019-02-28

**Authors:** Vanessa Nogueira Lages Braga, Camila de Carvalho Juanes, Hélio de Souza Peres, José Robson de Sousa, Bruno Coêlho Cavalcanti, Francisco Vagnaldo Fechine Jamacaru, Telma Leda Gomes de Lemos, Conceição Aparecida Dornelas

**Affiliations:** I Fellow Master degree, Postgraduate Program in Medical Surgical Sciences, School of Medicine, Universidade Federal do Ceará (UFC), Fortaleza-CE, Brazil. Intellectual and scientific content of the study, technical procedures, manuscript preparation.; II Fellow Master degree in Pathology, School of Medicine, UFC, Fortaleza-CE, Brazil. Technical procedures, responsible for propolis extraction.; III Graduate student, School of Medicine, UFC, Fortaleza-CE, Brazil. Technical procedures.; IV Graduate student, Faculdade Maurício Nassau, Fortaleza-CE, Brazil. Technical procedures.; V PhD, Department of Physiology and Pharmacology, National Laboratory of Experimental Oncology, UFC, Fortaleza-CE, Brazil. Biological assays.; VI PhD in Surgery, Researcher, Nucleus of Research and Development of Medicines, Laboratory of Pharmacology and Preclinical Research, School of Medicine, UFC, Fortaleza-CE, Brazil. Analysis and interpretation of data, statistics analysis.; VII PhD, Full Professor, Department of Organic and Inorganic Chemistry, UFC, Fortaleza-CE, Brazil. Analysis and interpretation of data, statistics analysis.; VIII PhD, Associate Professor, Postgraduate Program in Pathology and Medical-Surgical Sciences, School of Medicine, UFC, Fortaleza-CE, Brazil. Design of the study, critical revision, final approval.

**Keywords:** Propolis, Lysine, Gum Arabic, Azoxymethane, Colorectal Neoplasms, Rats

## Abstract

**Purpose:**

To evaluate red propolis, gum arabic and L-lysine activity on colorectal preneoplastic lesions induced by azoxymethane (AOM).

**Methods:**

The study featured 4 control groups (I-IV) and 4 experimental groups (V-VIII), totaling 48 rats. Once a week for 2 weeks, animals on control groups received saline, while animals in experimental groups received azoxymethane (15 mg/kg i.p.). The follow up along 16 weeks included daily oral gavage to administer water (I and V), L-lysine (150 mg/kg)(II and VI), própolis (100mg/5ml/kg)(III and VII), or gum arabic (5ml/kg)(IV and VIII). Was performed surgery on the animals in the end of this time in order to collect blood for biological assays (TBARS, GSH), followed by their sacrifice to tissue extract.

**Results:**

Oxidative stress (TBARS) and the number of aberrant crypt foci (ACF) in distal colon were lower using própolis (p<0.01 for both parameters). Gum arabic reduced preneoplastic lesions (ACF ≤ 4 crypts) on distal colon and on the entire colon (p<0.05).

**Conclusions:**

Red propolis reduced AOM-induced oxidative stress (TBARS) and total number of ACF in the distal colon. L-lysine neither protected against nor enhanced AOM-induced ACF. Gum arabic reduced the number of ACF.

## Introduction

 In Brazil, colorectal cancer (CRC) is the third-most frequent type of cancer among women and the fourth among men^1^. The use of preneoplastic lesions as biomarkers for CRC was pioneered by Bird who proposed a classification of aberrant crypt foci (ACF) based on a colon`s study of murine treated with azoxymethane (AOM)^2^.

 Red propolis is a balsamic resinous mixture of wax, saliva and vegetable exudate (derived from tree bark, leaf buds and pollen) produced by africanized bees (*Apis mellifera*)^3^. Brazilian red propolis contains a range of antioxidant compounds with promising carcino preventive properties^4^.^^

*L*-lysine is an essential amino acid for humans^5^. However, in animals with carcinomas induced by N-butyl-N-(4-hydroxybutyl)-nitrosamine, the coadministration of *L*-lysine promoted carcinogenesis in 100% of cases, when compared to carcinogen alone^6^.

 Gum arabic at 1% was used to dilute the red propolis from propolis *in natura*, which is water-insoluble. Some authors have found antioxidant and anti-inflammatory properties for this composition^7^.

 The purpose of this study was to assess actions of red propolis and L-lysine on experimental colorectal carcinogenesis based on the quantification of ACF under stereoscopic microscopy. We also evaluated oxidative stress (GSH and TBARS), genotoxicity (comet assay in peripheral blood and micronucleus assay in peripheral blood and bone marrow), and calculated the reticulocyte/erythrocyte ratio in peripheral blood.

 Due to the dizzying increase in cancer incidence and toxicity of its therapies, much effort has been invested in identifying new treatment with natural compounds. Studies published so far on Brazilian red propolis (PVB) and its isolated components have shown promising results in relation to free radical scavenging properties and ability to act as antioxidant biomarkers^8^.

## Methods

 The study protocol followed the guidelines of the Brazilian Society of Animal Experimentation (COBEA), and was approved by the Animal Research Ethics Committee (CEUA), Universidade Federal do Ceará (nº 17/2016).

**Figure 1:**
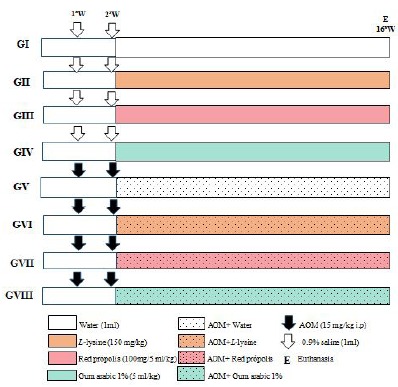
Study’s design showing substances administered, injection of AOM time and duration of the whole experiment.

 The study featured 4 control groups (Groups I-IV, n=5) and 4 experimental groups (Groups V-VIII, n=7), totaling 48 animals (Wistar rats weighing 40-60 g). Once a week, for 2 weeks, animals in control groups received saline while animals of experimental groups received AOM (15 mg/kg i.p.). In the next step, along 16 weeks all of them received daily through oral gavage water (Groups I and V), L-lysine (Groups II and VI), propolis (Groups III and VII), or gum arabic (Groups IV and VIII) (Fig. 1). At the end of this period, surgery was performed to collect blood sample, followed by euthanasia with an overdose of anesthetics to extract colon and bone marrow.

###  Carcinogen 

 Azoxymethane^®^ was purchased from Sigma-Aldrich Corporation as ampoules of 100mg and diluted in sterile saline solution to obtain a dose of 15mg / kg body weight. The solution was then injected intraperitoneally (i.p) in animals of Groups V through VIII once a week, for 2 weeks.

###  Red própolis 

 Propolis *in natura* was acquired from a trusted supplier in Barra de Santo Antônio (Alagoas). Because propolis is water-insoluble, it was diluted in gum arabic solution to 1% in water (100mg/5ml/kg - by gavage), following the protocol described by Shulka *et al.* ^9^, with adaptations.

###  L-lysine 

*L*-lysine monohydrochloride (C_6_H_14_N_2_O_2_·HCl; CAS #657-27-2) produced in China was diluted in water and administered intraperitoneally at a dose of 150 mg/kg body weight. The dilution was prepared once a day, at the time of injection. 

###  Gum arabic 1% 

 Gum arabic 1% a.g. (Dinâmica Química Contemporânea Ltda) was diluted in distilled water and administered by gavage at a dose of 5 mL/kg body weight. The dilution was prepared once a week, taking into account changes in body weight.

###  Surgical procedure 

 Following a 12-hour fast with access to water *ad libitum*, rats were anesthetized with intraperitoneal injection of ketamine (80 mg/kg) and xylazine (8 mg/kg). Animals had their abdomen shaved, they were placed in dorsal decubitus on sterilized drape and their paws were secured with tape. Then laparotomy was performed with a midline xyphopubic incision exposing the abdominal cavity and the retroperitoneal space. Blood was collected from the abdominal aorta and animals were sacrificed. After that, their colon were extracted up to the cecum. The colon was then opened along the antimesenteric border, rinsed and irrigated with saline, laid out on kraft paper and rolled up, followed by fixation in 10% buffered formaldehyde for 24 hours. 

 To perform the micronucleus assay in bone marrow the femur was excised, cleaned and had its proximal epiphysis sectioned. Bone marrow was extracted using thin needle in 1 ml syringes pre-filled with 0.5 ml of fetal bovine serum. The needle was firmly inserted into the opening of the femur and the fetal bovine serum was injected so as to move the marrow into a centrifuge tube containing 3 ml of that serum.

###  ACF evaluation 

**Figure 2:**
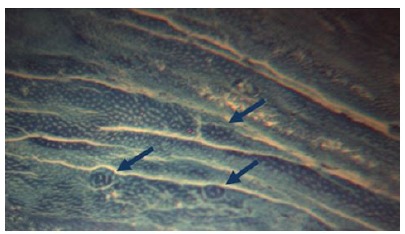
Aberrant crypt foci (*arrows*) in rat’s colon treated with AOM.

 After 24 hours of fixation, each colon specimen was immersed in 0.1% methylene blue and phosphate-buffered saline for 1 minute (Fig. 2). Under stereoscopic microscopy (M90 Vasconcellos, DF Vasconcellos S.A), the number of ACF and the number of crypts per focus were registered to determine distribution and multiplicity^2^ per colon segment (distal, middle and proximal). The colon mucosa was evaluated under stereoscopic microscopy at x40 magnification. Following crypt quantification, the specimen was rolled up in Kraft paper and preserved in 10% buffered formaldehyde. 

###  Biological assays 

#### Alkaline comet assay in peripheral blood

 The alkaline comet assay for assessing DNA damage was conducted as described by Singh *et al*.^10^, with slight modifications. 

#### Micronucleus assay in peripheral blood

 The micronucleus test was performed using the acridine orange staining technique to analyze the frequency of micronuclei in peripheral blood reticulocytes, according to Hayashi *et al.* ^11^.

#### Micronuclei in bone marrow cells

 The bone marrow was extracted injecting fetal bovine serum - FBS into femur medullary cavity to displaced it into a centrifuge tube containing 3 mL FBS. The harvested bone marrow cells were centrifuged for 5 minutes at 1000 rpm, the supernatant was discarded and the precipitate was homogenized. A drop of cell suspension was placed on a clean and dry slide for smearing. Two slides were prepared for each animal. The material was then stained with the Leishman technique and analyzed under a binocular optic microscope at x20 and x40 magnification, using the criteria of Schmid^12^ for the identification of micronuclei. For each set of two slides, 1000 polychromatic erythrocytes (PCE) were analyzed. The cytotoxicity of the treatment was proxied by the ratio between PCE and normochromatic erythrocytes (NCE) based on 1000 erythrocytes (PCE or NCE) per animal^13^.

###  Oxidative stress 

#### Dosage of glutathione (GSH)

 The 3 mL peripheral blood collected from the anesthetized animals was centrifuged and frozen in liquid nitrogen at -70⁰C to determine the GSH concentration (method of Sedlak)^14^. The reaction product was quantified with a Beckman spectrophotometer at 412 nm**.**

#### Dosage of the concentration of thiobarbituric acid substances (TBARS) 

 Using peripheral blood, lipid peroxidation was quantified with the TBARS Assay^15^. The blood was subsequently centrifuged and the plasma immediately stored in liquid nitrogen at -70⁰C.

#### Reticulocyte/erythrocyte ratio

 Acridine orange dye was used to count reticulocytes in peripheral blood, as described by Hayashi *et al*.^11^. The result was expressed as percentage of the total number of erythrocytes in the sample.

###  Statistical analysis 

 Having confirmed the normality of distribution of the quantitative variables with the Shapiro-Wilk test in the vast majority of cases, descriptive statistics were performed by calculating mean values and standard deviations, while parametric tests were used for analytical statistics. Pairwise comparisons between the four substances (water, *L*-lysine, propolis, gum arabic) were made with one-way ANOVA associated with Tukey’s multiple comparison test for both control groups and experimental groups. Using the *t* test for unpaired samples, the control groups were then compared to the experimental groups (saline vs. AOM) for each substance.

 The software GraphPad Prism v. 7.00 (GraphPad Software, La Jolla, California, USA) was used for both analysis and graphing. All tests were two-tailed, with the level of statistical significance at 5% (*p*<0.05).

## Results

###  Variation in body mass 

 Percentage weight gain was determined for the four experimental groups (Group V: 214.51 ± 26.67%; Group VI: 210.41 ± 50.58%; Group VII: 231.09 ± 51.29%; Group VIII: 189.76 ± 27.70%), but the observed differences were nonsignificant (ANOVA associated with Tukey: *F*=1.2089; *p*=0.3298).

###  ACF count (total and per segment) 

**Figure 3:**
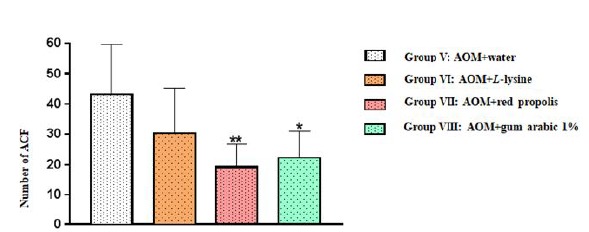
Number of aberrant crypt foci (ACF) in the distal colon segment of the animals in the four experimental groups. The results are expressed as mean values and standard deviation.

 In all the four experimental groups, the total number of ACF was statistically similar in the proximal and middle colon segments, but a significant difference was observed for the distal segment: Group VII (AOM+ propolis) and Group VIII (AOM+ gum arabic) displayed significantly fewer ACF than Group V (AOM+ water) (**p=0.0096 and *p=0.0250, respectively) (Table 1, Fig. 3). 

**Figure 4:**
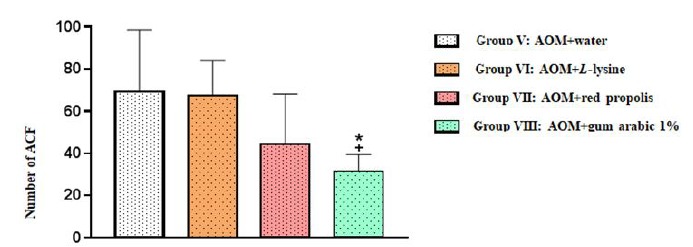
Number of aberrant crypt foci (ACF) in the entire colon of the animals in the four experimental groups. The results are expressed as mean values and standard deviation.

 When the entire colon was considered, Group VIII (AOM+ gum arabic) displayed significantly fewer ACF than Group V (AOM+ water) (*p=0.0166) and Group VI (AOM+*L*-lysine) (^+^ *p*=0.0237). The ACF count was also reduced in Group VII (AOM+ propolis), but the difference was nonsignificant (ANOVA associated with Tukey’s multiple comparison test) (Fig. 4).

 Compiling up all data observed along the entire length of the three colonic segments, as a whole, we can build the following table (Table 1).

**Table 1:** Number of aberrant crypt foci (ACF) in the entire colon and in each segment.

Colon segment	Group V (water)	Group VI (L-lysine)	Group VII (propolis)	Group VIII (gum arabic)	p-value (ANOVA)
Proximal	2.33 ± 3.83	0.00 ± 0.00	2.29 ± 2.63	0.00 ± 0.00	0.1152
Middle	23.83 ± 18.00	37.17 ± 20.13	22.71 ± 17.63	9.29 ± 8.12^c^	0.0462
Distal	43.17 ± 16.46	30.33 ± 14.90	19.29 ± 7.46^a^	22.14 ± 8.88^b^	0.0093
Entire colon	69.33 ± 29.21	67.50 ± 16.72	44.29 ± 23.89	31.43 ± 8.02^b, c^	0.0077

ANOVA=analysis of variance; ^a^=statistically significant in relation to Group V (*p*<0.01); ^b^=statistically significant in relation to Group V (*p*<0.05); ^c^=statistically significant in relation to Group VI (*p*<0.05) (Tukey test). Significant *p*-values are in bold type.

###  ACF with ≤ 4 crypts (total and per segment) 

**Figure 5:**
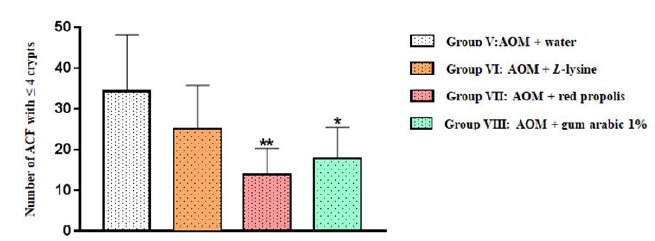
Number of aberrant crypt foci (ACF) with ≤ 4 crypts in the distal colon segment of the animals in the four experimental groups. The results are expressed as mean values and standard deviation.

 No significant difference was observed between the four experimental groups with regard to ACF with ≤ 4 crypts in the proximal and middle colon segments (ANOVA associated with Tukey’s multiple comparison test), but a significant difference was observed for the distal segment: multiplicity was significantly lower in Group VII (AOM+ propolis) (*p*=0.0060) and Group VIII (AOM+ gum arabic) (*p*=0.0295) in relation to Group V (AOM+ water) (Fig. 5, Table 2). 

**Table 2:** Number of aberrant crypt foci (ACF) with ≤ 4 crypts in the entire colon and in colon segments (distal, middle, proximal) of the animals in the four experimental groups. Results are expressed as mean values and standard deviation.

Colon segment	Group V (water)	Group VI (L-lysine)	Group VII (propolis)	Group VIII (gum arabic)	p-value (ANOVA)
Proximal	2.17 ± 3.71	0.00 ± 0.00	2.14 ± 2.41	0.00 ± 0.00	0.1250
Middle	18.33 ± 13.53	31.17 ± 16.74	13.00 ± 10.91	6.71 ± 5.71^c^	0.0111
Distal	34.33 ± 13.87	25.00 ± 10.81	13.86 ± 6.4^a^	17.71 ± 7.70^b^	0.0065
Entire colon	54.83 ± 24.79	56.17 ± 11.51	29.00 ± 16.29b^d^	24.43 ± 7.66b^c^	0.0018

ANOVA=analysis of variance; ^a^=statistically significant in relation to Group V (*p*<0.01); ^b^=statistically significant in relation to Group V (*p*<0.05); ^c^=statistically significant in relation to Group VI (*p*<0.01) ^d^=statistically significant in relation to Group VI (*p*<0.05) (Tukey test). Significant *p*-values are in bold type.

**Figure 6:**
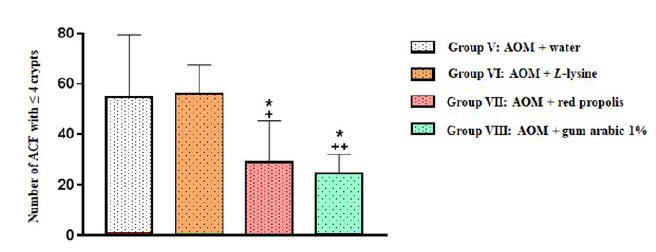
Number of aberrant crypt foci (ACF) with ≤ 4 crypts in the entire colon of animals in all four experimental groups. Results are expressed as mean values and standard deviation.

 When the entire colon was considered, Group VII (AOM+ propolis) displayed significantly fewer ACF with ≤ 4 crypts than Group V (AOM+ water) (*p*=0.0394) and Group VI (AOM+ *L*-lysine) (*p*=0.0285). A similar pattern was observed for Group VIII (AOM+ gum arabic) in relation to Group V (AOM+ water) (*p*=0.0127) and Group VI (AOM+ *L*-lysine) (*p*=0.0090) (Fig. 6, Table 2).

 Consolidating the statistical mean and standard deviation between the groups submitted to the AOM, the number of ACF have up to 4 crypts verified in several segments, as well as throughout the colonic segment (Table 2).

###  ACF with ≥ 5 crypts (total and per segment) 

 No significant difference was observed between the four experimental groups with regard to ACF with ≥ 5 crypts, whether the entire colon or specific segments were considered.

###  Alkaline comet assay and micronucleus assay in reticulocytes in peripheral blood 

 The frequency of damaged DNA or micronucleated peripheral blood cells (lymphocytes) was statistically similar between groups. In other words, at the dose and time of exposure used in the study, AOM produced no measurable genotoxic or mutagenic effects.

###  Micronucleus assay in bone marrow 

 The experimental groups did not differ significantly with regard to genotoxicity in the micronucleus assay in bone marrow. However, were observed genotoxicity damages in the Group VI (AOM+ *L*-lysine) and Group VIII (AOM+ gum arabic), in regard of their respective controls, L-Lysine (Group II) (p = 0.0162) and Gum arabic (Group IV) (p = 0.0404).

###  GSH in peripheral blood 

 GSH measurements were statistically similar for all eight study groups. 

###  TBARS in peripheral blood 

 Lipid peroxidation was significantly more intense in all experimental groups, but the increase was significantly smaller in Group VII (AOM+ propolis) than in Group V (AOM+ water), VI (AOM+ *L*-lysine) and VIII (AOM+ gum arabic)(Table 3).

**Table 3:** TBARS levels in peripheral blood of animals receiving saline (control groups) or azoxymethane (experimental groups). Results are expressed as mean values and standard deviation.

Exposed to AOM	Water	L-lysine	Propolis	Gum arabic	p-value (ANOVA)
No	12.60 ± 2.79	9.60 ± 2.97	12.00 ± 2.55	10.60 ± 3.21	0.3772
Yes	32.17 ± 7.99	31.67 ± 3.20	22.00 ± 5.10^a,b^	32.71 ± 4.99	0.0043
*p*-value (*t* test)	0.0006	<0.0001	0.0025	<0.0001	

AOM=azoxymethane; ANOVA=analysis of variance; ^a^=statistically significant in relation to Group V and Group VI (*p*<0.05); ^b^=statistically significant in relation to Group VIII (*p*<0.01) (Tukey test). Significant *p*-values are in bold type.

###  Reticulocyte/erythrocyte ratio in peripheral blood 

 The reticulocyte/erythrocyte ratio (a proxy for cell turnover) was significantly lower in experimental groups, especially in Group VII (AOM+ propolis) *p*= 0.0009, followed by Group V (AOM+ water) *p=* 0.0074 and Group VIII (AOM+ gum arabic) *p*= 0.0084. However, when comparing the four experimental groups, differences were nonsignificant.

## Discussion

 Throughout the 16-week experiment, variation in body weight was statistically similar in all eight study groups, indicating that none of the tested substances interfered with food consumption or weight gain. Our finding matches the results of another study evaluating weight gain in rats exposed to AOM^16^. 

 Using a stereoscopic microscope, we quantified classical ACF, ACF ≤ 4 crypts, and ACF ≥ 5 crypts (multiplicity). No polyps were visible. No ACF were observed in the control groups (Groups I-IV).

 The fact that significantly fewer ACF were observed in the distal colon segment in Group VII (AOM+ própolis)(*p*=0.0096) and Group VIII (AOM+ gum arabic) (*p*=0.0250) than in Group V (AOM+ water) suggests propolis and gum arabic had protective activity, possibly associated with the putative antioxidant properties of these two substances^8^ ^,^ ^17^.

 When the colon was considered as a whole, the total number of ACF was significantly smaller in Group VIII (AOM+ gum arabic) than in Group V (AOM+ water) (*p*=0.0166). In fact, both gum arabic and propolis had the effect of reducing the total number of ACF in the colon, but the difference was nonsignificant for Group VII (AOM+ própolis). 

 When interpreting the results for gum arabic, it should be kept in mind that the animals in Group VII also received a certain amount of gum arabic since this substance was employed in the dilution of red propolis. However, apart from its documented ability to capture free radicals, the chemical composition of Brazilian red propolis has not yet been fully mapped^18^. Like many natural products, it features a complex array of vegetable and animal components, some of which may have inhibited the beneficial effects of the gum. Moreover, several compounds are known to be both antioxidant and pro-oxidant depending on the dose administered and the amount absorbed. Thus, interference, if any, may have occurred at the level of absorption or action, or both.

 Earlier studies have shown that ACF are unevenly distributed in the colon, with most developing in the middle and distal segments^19^. In the DMH/AOM rat model, tumors develop most frequently in the distal colon and least frequently in the proximal colon, possibly due to deficiencies in DNA repair mechanisms^20^. Our findings agree with the literature since most ACF were found in the distal colon, with no interference from the test substances (red propolis, *L*-lysine, gum arabic 1%) on ACF distribution or multiplicity.

 When the distal colon segment was evaluated for ACF ≤ 4 crypts, the number of ACF was significantly smaller in Group VII (AOM+ propolis) (*p*=0.0060) and Group VIII (AOM+gum arabic) (*p*=0.0295) than in Group V (AOM+ water), suggesting a protective effect. When the entire colon was evaluated for ACF ≤ 4 crypts, the number of ACF was also significantly smaller in Group VII and Group VIII than in Group V (*p*=0.0394 and *p*=0.0127, respectively). 

 Bird^21^ proposed to quantify ACF along each colon segment, as ACF with ≥ 5 crypts are more likely to persist and increase in size, their presence may be used as a predictor of carcinogenesis.^ ^ In this way Boateng^22^, sorted out three multiplicity grade: small (1-3 crypts per focus), medium (4-5 crypts per focus) and large (≥5 crypts per focus), apparently not perceiving that the ones with exactly 5 crypts per focus were counted repeatedly in both groups, the medium and the largest one. Adapting his work, and trying to highlight differences, even more as we were working with lower AOM doses, sufficient only to promote preneoplastic lesions, we modified this design considering only two groups: the first one with 4 or less crypts per foci, and the second one with 5 or larger number of crypts per foci.

 In the evaluation of ACF ≥ 5 crypts, all four experimental groups displayed no significant differences, regardless of the colon segment considered. Unfortunately, our study design (dose and time of exposure) did not allow exploring this interesting pattern of ACF multiplicity. 

 Crypt hyperplasia is known to precede CRC, but in experimental models the earliest stage observed is the appearance of dysplastic crypts or foci of dysplasia in the colon mucosa^23^. In the present study, ACF were most abundant and ACF multiplicity was greatest in the distal and middle colon segments of rats exposed to AOM, matching the findings of earlier studies.

 AOM-induced oxidative stress was proxied by GSH and TBARS levels in peripheral blood. The lack of variation in GSH levels across the study groups suggests this antioxidant was not being consumed prior to euthanasia. On the other hand, TBARS levels rose significantly in all groups exposed to AOM, although the increase was significantly less dramatic in Group VII (AOM+ propolis) than in the other three groups, suggesting a protective effect against oxidative stress. The latter appears to contribute to ACF formation in the colon as well as the hepatic genotoxicity seen in our study (unpublished data). Red propolis is reported to have considerable antioxidant and antibacterial properties, and some subfractions may be more biologically active than crude extract^9^.

 According to some authors, genotoxicity may be related to the formation of reactive oxygen species, which can cause severe intracellular oxidative stress through changes in macromolecules such as oxidated lipids, proteins and DNA^24^.

 All chemical carcinogenic initiators are electrophilic and contain highly reactive electron-deficient species capable of binding to nucleophilic sites, damaging the DNA directly, triggering mutation and fostering cancer^25^. AOM is widely used to induce CRC in rat models through oxidative stress-dependent mechanisms^26^. Oxidative stress is reported to induce the formation of lipid peroxides and other ROS and thus plays a central role in carcinogenesis^27^.

 The comet assay and the micronucleus assay were used to evaluate AOM-induced genomic and cytogenetic damage, respectively. However, at the dose employed, no significant difference in genotoxicity or mutagenicity in peripheral blood (lymphocytes) was observed between the experimental groups. Chemopreventive compounds may act by chelating/inactivating mutagens, thereby preventing damage to the DNA (desmutagens), or by interfering with cellular fixation processes associated with DNA repair (bio-antimutagens)^28^. Splenic phagocytosis of cells with signs of genotoxicity, as observed by Ishi *et al.* ^29^, may also have to be taken into account.

 The experimental groups did not differ significantly with regard to genotoxicity in the comet assay in peripheral blood, but Group VI (AOM+ *L*-lysine) differed significantly from Group II (*L*-lysine) (*p*=0.0284). According to a recent study on oral cancer, DNA damage or micronuclei in oral mucosa cells or in peripheral blood are indications (or markers) of recent exposure to noxious physical, chemical or environmental agents^30^. In our study, the evaluation of DNA damage was performed 17 weeks after exposure to the carcinogen. In the assay detecting AOM-induced genotoxicity in bone marrow, the experimental groups displayed statistically similar results. 

 The reticulocyte/erythrocyte ratio was lower in the experimental groups than in the control groups, indicating fewer reticulocytes and less cell damage, hence smaller cell turnover. This was particularly evident in Group V (AOM+ water), Group VII (AOM+ propolis) and Group VIII (AOM+ gum arabic), with emphasis on Group VII. Propolis apparently reduced cell damage (thus, the need for cell renewal), or it may have inhibited proliferation. On the other hand, while fewer reticulocytes were formed in the four experimental groups, the reticulocyte/erythrocyte ratio was statistically similar between them. In this case, the effect observed at the discrete functional and medullary level is probably due to AOM inhibiting proliferation.

## Conclusions

 Based on the total number of ACF and the number of ACF ≤ 4 crypts (preneoplastic lesions) in the distal colon and the entire colon, red propolis reduced pre neoplastic lesions in rats. *L*-lysine neither protected against nor enhanced AOM-induced colorectal carcinogenesis. Red propolis and *L*-lysine displayed no protective effect against genotoxicity and mutagenesis at the dose and time tested. AOM invariably caused oxidative stress, reduced by red propolis. Gum arabic 1% reduced the number of ACF, protecting the colon.
